
JOURNAL INFORMATION
==============================
NLM Title Abbreviation: ScientificWorldJournal
Journal ID: ScientificWorldJournal
NLM Journal ID: 101131163
Journal ID: TSWJ

The Scientific World Journal

ISSN: 2356-6140
EISSN: 1537-744X
Publisher: Wiley

ARTICLE INFORMATION
==============================
PMCID: PMC6009634
PMID: 29973800
DOI: 10.1100/tsw.2002.26
Article ID: 965176
Article version: 1
Subjects: Short Report

Engineering Monomeric Glycogenin

Dwek David
Fisher David
Lomako Joseph
Lomako Weislawa M.
Whelan William J.
Department of Biochemistry and Molecular Biology, University of Miami School of Medicine, P.O. Box 016129 (M823), Miami, FL 33101, USA
Publication date: 2002
Electronic publication date: 2002 Mar 8
Volume: 2
First page: 51
Last page: 52
Copyright: Copyright © 2002 David Dwek et al.
Copyright year: 2002
License: This is an open access article distributed under the Creative Commons Attribution License, which permits unrestricted use, distribution, and reproduction in any medium, provided the original work is properly cited.
License URL: https://creativecommons.org/licenses/by/3.0/

==============================
Advances in Gene Technology: The Genome and Beyond –
Structural Biology for Medicine (Proceedings of the 2002 Miami
*Nature Biotechnology* Winter Symposium)
*TheScientificWorld* 2002, 2(S2), 51–52
ISSN 1532-2246; DOI 10.1100/tsw.2002.26

### ENGINEERING MONOMERIC GLYCOGENIN

David Dwek, David Fisher, Joseph Lomako, Weislawa M. Lomako, and William J. Whelan
Department of Biochemistry and Molecular Biology, University of Miami School of Medicine,
P.O. Box 016129 (M823), Miami, FL 33101

**INTRODUCTION.** Glycogenin is an autocatalytic, self-glucosylating muscle protein that is the primer for glycogen synthesis. It has Mr 37kDa and constructs a maltosaccharide chain on its Tyr 194, the chain in turn being elongated by glycogen synthase. UDPglucose is the substrate for both enzymes[1]. There is an obvious interest in the two-dimensional structure of glycogenin, but attempts by a colleague to crystallize the enzyme have proven unsuccessful. We observed that recombinant glycogenin contained oligomers, apparently formed via disulfide bonds between various of its four cysteine residues. The glycogenin did not contain free sulfhydryl groups, suggesting that all the cysteines were involved in intra- or intermolecular disulfide bonds. Splitting of the intermolecular bonds by thiol reagents only took place when the protein was heated. Accordingly, to obtain monomeric glycogenin we have constructed a series of mutants in which the cysteines were replaced by serine.

**MATERIALS AND METHODS.** Wild-type rabbit muscle glycogenin was expressed and mutagenized as by Alonso et al.[2]. Activity measurements and Western blotting were carried out as by Lomako et al.[3].

**RESULTS AND DISCUSSION.** Glycogenin has Cys residues at positions 88, 97, 130, and 270[4]. All possible single, double, triple, and quadruple mutations were constructed and expressed. The extracts were tested for (1) activity by self-glucosylation with UDP[$^{14}$C]glucose, (2) level of expression, by Western blotting, and (3) whether oligomers were noted, by SDS-PAGE, directly, and by radioautography, after [$^{14}$C]-glucosylation.

The levels of activity and expression in the extracts are recorded qualitatively in Table 1, relative to what was seen with wild-type glycogenin, expressed under the same conditions.

A wide range of activities and levels of expression was seen, ranging from no expression, through poor expression and activity to levels comparable with those of wild-type enzyme. It is noteworthy that while all the single Cys mutants still formed oligomers, none of the double, triple or quadruple mutants did so, save that no expression of the 88 + 97 + 130 mutant was seen.

Several of the multiple mutants are candidates for crystallization of their glycogenins, when oligomerization, if that is a factor in inhibiting crystallization, will not interfere.

TABLE 1

Mutation of Cysteine Residues to Serine in Recombinant Rabbit-Muscle Glycogenin

| Position of Mutation | Activity | Expression | Oligomerization |
|---|---|---|---|
| Single Mutants | | | |
| 88 | ++ | ++ | Yes |
| 97 | +++++ | +++++ | Yes |
| 130 | ++ | +++ | Yes |
| 270 | +++++ | +++ | Yes |
| Double mutants | | | |
| 88+97 | +++ | +++ | No |
| 88+130 | No | + | No |
| 88+270 | ++++ | +++ | No |
| 97+130 | ++ | +++ | No |
| 97+270 | +++++ | ++++ | No |
| 130+270 | No | ++ | No |
| Triple mutants | | | |
| 88+97+130 | No | No | No |
| 88+97+270 | ++ | ++ | No |
| 88+130+270 | ++ | +++ | No |
| 97+130+270 | No | + | No |
| Quadruple mutants | | | |
| 88+97+130+270 | No | ++ | No |

The results are presented relative to wild-type glycogenin, which was assigned 6+.

Supported by a grant from NIH, DK 37500.

**REFERENCES**

1. Alonso, M.D. Lomako, J., Lomako, W.M., and Whelan, W.J. (1995) FASEB J. 9, 1126–1127.
2. Alonso, M.D., Lomako, J., Lomako, W.M., and Whelan, W.J. (1994) FEBS Lett. 342, 38–42.
3. Lomako, J., Lomako, W.M., and Whelan, W.J. (1988) FASEB J. 2, 3097–3103.
4. Viscupic, E., Cao, Y., Zahng, W., Chag, C., DePaoli-Roach, A.A., and Roach, P.J. (1992) J. Biol. Chem. 267, 25759–25763.
